# Explainable deep learning approach for extracting cognitive features from hand-drawn images of intersecting pentagons

**DOI:** 10.1038/s41746-023-00904-w

**Published:** 2023-08-23

**Authors:** Shinya Tasaki, Namhee Kim, Tim Truty, Ada Zhang, Aron S. Buchman, Melissa Lamar, David A. Bennett

**Affiliations:** 1 Rush Alzheimer’s Disease Center, Rush University Medical Center, Chicago, IL USA; 2 Department of Neurological Sciences, Rush University Medical Center, Chicago, IL USA; 3 Department of Psychiatry and Behavioral Sciences, Rush University Medical Center, Chicago, IL USA

**Keywords:** Biomarkers, Neurological disorders

## Abstract

Hand drawing, which requires multiple neural systems for planning and controlling sequential movements, is a useful cognitive test for older adults. However, the conventional visual assessment of these drawings only captures limited attributes and overlooks subtle details that could help track cognitive states. Here, we utilized a deep-learning model, PentaMind, to examine cognition-related features from hand-drawn images of intersecting pentagons. PentaMind, trained on 13,777 images from 3111 participants in three aging cohorts, explained 23.3% of the variance in the global cognitive scores, 1.92 times more than the conventional rating. This accuracy improvement was due to capturing additional drawing features associated with motor impairments and cerebrovascular pathologies. By systematically modifying the input images, we discovered several important drawing attributes for cognition, including line waviness. Our results demonstrate that deep learning models can extract novel drawing metrics to improve the assessment and monitoring of cognitive decline and dementia in older adults.

## Introduction

Copying geometric or abstract figures is a complex behavior that requires the integration of multiple cognitive domains, including executive function to initiate the task, visuospatial abilities to carry it out, and, to a lesser extent, semantic memory to construct the correct image. Therefore, paper-and-pencil drawing tasks are often employed independently or as part of a larger screening tool to detect cognitive impairment, including Alzheimer’s dementia^1,2^, and Parkinson’s disease^3,4^. For example, copying intersecting pentagons, the Pentagon Drawing Test (PDT), is used as one of the items in the 30-item Mini-Mental State Examination (MMSE) - a common tool to evaluate a person’s mental health and identify potential cognitive impairments^5^. PDT involves asking the participants to draw two intersecting pentagons on a piece of paper. In the MMSE, the intersecting pentagons are rated simply 0 (fail) or 1 (pass) based on limited factors. More detailed evaluations of the intersecting pentagons have been shown to provide granular-level information about a person’s cognitive abilities and potential dementia status^1,6^.

While paper-and-pencil drawing tests, like the PDT, can be a useful tool in assessing a person’s cognitive abilities, the conventional scoring has some limitations. First, these tests are often prone to subjective assessment and rater bias with different raters potentially having different interpretations of the drawings, which can lead to inconsistencies in the scoring and potentially impact the accuracy of the results. Secondly, these tests can be labor-intensive and time-consuming to score, particularly when used within a larger population. Finally, these tests are typically focused on a limited set of drawing attributes. For example, the PDT scoring is based on only a few factors, such as the presence or absence of intersections and the number of vertices^5^, which only capture limited aspects of behavior and hence cognitive status^1^. Thus, while paper-and-pencil drawing tests can be useful, their human-based scoring has limitations in scope, accuracy, and scalability.

Automated scoring methods that utilize machine learning methods offer the potential to address some or all of these weaknesses. For example, deep-learning techniques have demonstrated promising performance in automating ratings for the PDT^7,8^, Rey Complex Figure Test^9,10^, and Clock Drawing Test^11,12^. However, the primary objective of the previous automated scoring is to reproduce human-based conventional ratings, and few machine learning approaches directly predict cognitive performance from drawings^13^. In addition, prior work has not been able to explore the factors, such as motor abilities and brain pathologies, that may confound or mediate the specific facets of cognition when the construction of intersecting pentagons is impaired. Identifying such integrated mechanisms requires drawing images, various phenotypes, and multiple brain pathologies assayed in the same set of individuals and the analytical frameworks to integrate these data. Furthermore, none of the studies utilized the models to discover the novel drawing features associated with cognitive impairment, which may be important for advancing our understanding of visuospatial memory ability and motor coordination of drawing in older adults.

**Fig. 1:**
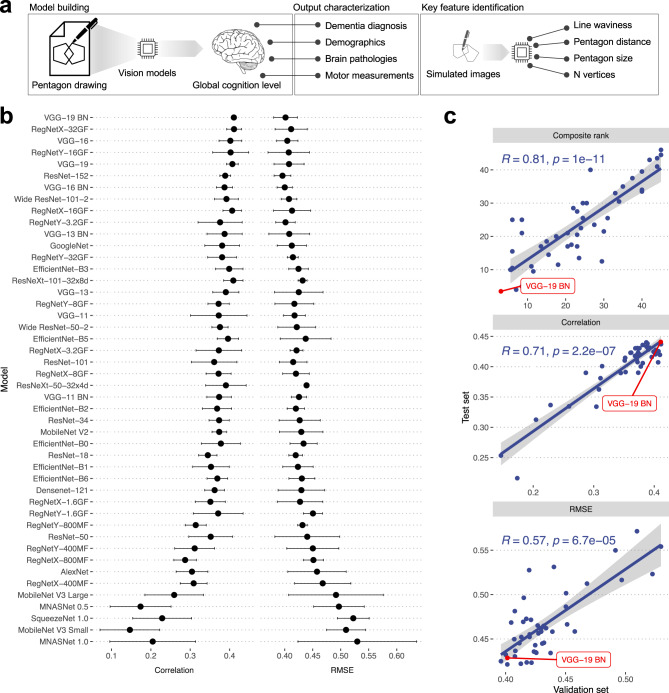
Development of a deep learning model for predicting global cognitive performance. **a** Schematic representation of the model development and interpretation process for predicting global cognition performance. The process includes (1) Model Building: selection of a high-performing vision recognition architecture from 47 established deep-learning models, (2) Output characterization: relations with comprehensive motor examinations and neuropathologies, and (3) Key feature identification: development of a pentagon-drawing simulator to identify key drawing characteristics in individuals with lower cognitive function. **b** Validation performances of the 47 deep learning models. Spearman’s correlation and RMSE between predicted cognition scores from each model and measured values were computed for validation samples. We repeated model training five times, each time using a distinct training set. The median and median absolute deviation of the metrics from the five runs was plotted. Models were ranked based on the average ranking of Spearman’s correlation and RMSE. **c** Comparison of model’s performances between validation and test sets. The composite ranking was obtained as an average of rankings based on Spearman’s correlation and RMSE. The composite ranking, Spearman’s correlation, and RMSE are compared between validation (x-axis) and test (y-axis) sets. The based on the VGG-19 BN architecture is highlighted as it showed the best permanence with the validation sets.

Here, we leveraged data from 3111 participants from three ongoing cohort studies of aging and dementia at the Rush Alzheimer’s Disease Center (RADC) to train a deep learning model that predicts global cognition performance (Fig. 1a). By performing a thorough evaluation of 47 established deep-learning models for vision recognition, we identified an architecture that demonstrated high and robust performance. Along with detailed cognitive tests, we were also able to incorporate comprehensive motor examinations conducted with participants and a variety of neuropathologies recorded for several hundred of participants. We used these data to break down the non-cognitive and pathological components of the model’s prediction. Furthermore, to address the challenge of interpretability of deep learning models, we developed a pentagon-drawing simulator. This simulator is an explainable-AI approach that allowed us to interrogate the deep learning model to suggest the key drawing characteristics in people with lower cognitive function.

## Results

### Characteristics of study participants

Participants enrolled at the age of 77.4 (SD: 7.6) with a follow-up of 5.6 years (SD: 4.6) (Table 1), on average. Of participants, non-Latino white (77.6%) is the most common ethnicity, followed by African American (21.6%). At yearly home visits, participants received comprehensive cognitive assessments, which included 19 tests used to generate a composite measure of global cognition. In addition, the PDT was administered as one of the items of the standard MMSE. Note that the PDT is not included in global cognition which is based on 19 measures independent of MMSE. We scanned 13,777 drawings of intersecting pentagons obtained throughout the visits.

**Table 1:** Demographic information for the ROS, MAP, and MARS cohorts.

	ROS	MAP	MARS
Number of participants	1093	1508	510
Number of samples	5575	6849	1353
Ethnicity (%)			
American Indian or Alaska Native	5 (0.5)	3 (0.2)	0 (0.0)
Asian	1 (0.1)	6 (0.4)	0 (0.0)
Black or African American	90 (8.2)	82 (5.4)	510 (100.0)
Native Hawaiian or Other Pacific Islander	1 (0.1)	1 (0.1)	0 (0.0)
Unknown	3 (0.3)	2 (0.1)	0 (0.0)
White	993 (90.9)	1414 (93.8)	0 (0.0)
Female (%)	777 (71.1)	1110 (73.6)	381 (74.7)
Education year (mean (SD))	18.18 (3.34)	14.61 (3.20)	14.93 (3.52)
Age at enrollment (mean (SD))	75.38 (7.28)	80.00 (7.32)	73.64 (6.29)
Age at visit (mean (SD))	79.52 (6.85)	82.11 (7.37)	75.58 (6.24)
Follow-up year (mean (SD))	8.29 (5.21)	4.33 (3.45)	3.37 (3.00)
Demented (%)	278 (25.4)	250 (16.6)	26 (5.1)
Global cognitive function (mean (SD))	−0.01 (0.73)	−0.11 (0.73)	−0.13 (0.57)
MMSE (mean (SD))	27.25 (3.23)	26.94 (3.39)	27.68 (2.27)
No cognitive impairment (NCI) (%)	579 (53.0)	896 (59.4)	386 (75.7)
Mild cognitive impairment (MCI) (%)	243 (22.2)	370 (24.5)	103 (20.2)
MCI, multiple causes (%)	11 (1.0)	14 (0.9)	1 (0.2)
Alzheimer’s dementia (Probable AD) (%)	218 (19.9)	196 (13.0)	18 (3.5)
Alzheimer’s dementia, multiple causes (Possible AD) (%)	28 (2.6)	17 (1.1)	0 (0.0)
Other dementia (%)	12 (1.1)	8 (0.5)	1 (0.2)
Vascular dementia (%)	46 (4.2)	39 (2.6)	6 (1.2)
Pentagon score = 1 (%)	509 (47.1)	822 (55.1)	307 (60.2)
Deceased (%)	769 (70.4)	924 (61.3)	155 (30.4)

### Model architecture for predicting cognition via drawing (PentaMind)

To develop models that predict global cognition from intersecting pentagon drawings, we comprehensively evaluated 47 published convolutional neural network models for image recognition (Methods). The 47 models were designed and trained to categorize images into 1000 object classes^14^. We modified the model structures so that the final output layer outputs a numeric value instead of object classes. As we collected multiple images from the same participant across the period, we used a person-based split rather than an image-based split to ensure that models were not trained with images from the same participants in both the training and validation/test sets. Specifically, we randomly divided 3111 participants into a training set (80%), a validation set (10%), and a test set (10%). To improve the reliability of performance evaluation, we repeated the training procedure five times using five distinct sets of training and validation samples.

Figure 1b indicates the comparative performance of the models based on Spearman’s correlation and root mean squared error (RMSE) for the validation sets. We selected a model that demonstrated superior performance in both correlation and RMSE metrics for further investigation. Specifically, we calculated a composite ranking as an average of the rankings based on Spearman’s correlation and RMSE. The model that ranked highest overall among the 47 models was the model based on VGG19 with batch normalization (VGG19-BN), which we named PentaMind. This model ranked second in Spearman’s correlation (0.41) and fourth in RMSE (0.40). The accuracy of PentaMind on the test sets was reasonably high, with a Spearman’s correlation of 0.44 and an RMSE of 0.42. Importantly, the performances of models on validation and testing sets are highly congruent, indicating that selecting a representative model based on validation sets did not introduce bias (Fig. 1c).

### Evaluation of PentaMind using test dataset

To conduct a series of evaluations for our PentaMind, we predicted the global cognition from all 13,777 images. However, applying the model to training samples may introduce bias into the estimates. To prevent this, we retrained PentaMind using an out-of-fold prediction strategy. Specifically, we divided the images into ten non-overlapping folds. Then, we trained the model using nine folds and tested it on the left-out tenth fold. This procedure was repeated for each of the ten folds, resulting in ten models, each trained on different sets of training and holdout images. By concatenating the predicted global cognitive function for the holdout samples, we obtained an unbiased prediction for all 13,777 images, which could then be used to calculate overall prediction accuracy and examine the characteristics of our prediction (Supplementary Fig. 1).

**Fig. 2:**
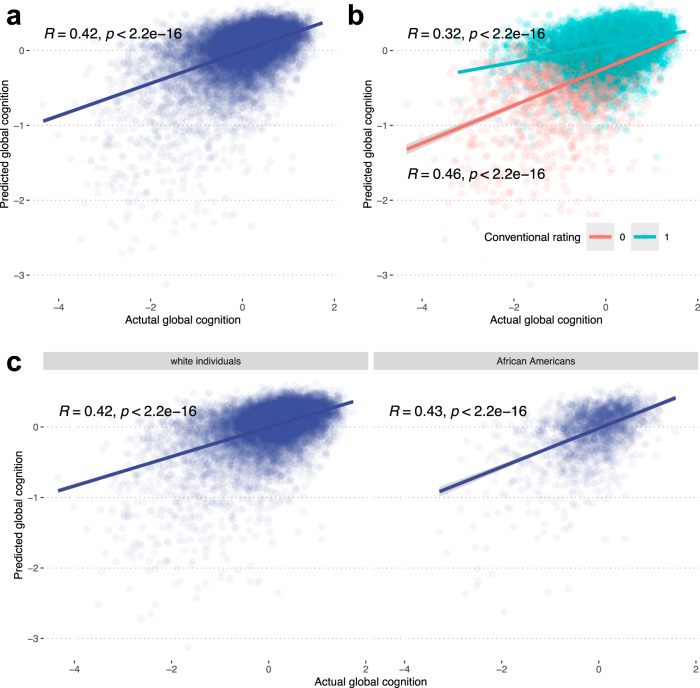
Performance evaluation of PentaMind. **a** Relationship of the predicted cognition scores and actual scores. We employed an out-of-fold prediction strategy to generate an unbiased predicted cognition score for all 13,777 images. The scatter plot compares the actual and predicted cognition scores with a linear regression line. Spearman’s correlation and *p*-value are also displayed. **b** PentaMind’s performance stratified by the conventional binary rating. The images are stratified based on the conventional rating: success (1) or failure (0). Spearman’s correlation and *p*-value for each group are displayed. **c** PentaMind’s performance stratified by the ethnicity of participants. The performance metric is calculated for white individuals and African Americans separately.

The predicted cognition score explained 23.3% of the variance in global cognition, which is 1.92 times greater than that of manual standard binary scoring (Fig. 2a and Supplementary Table 1) that is based on the presence or absence of intersections and the number of vertices. A regression model using PentaMind-predicted scores and human binary scores as covariates was able to explain 24.7% of the variance in global cognition together. Notably, PentaMind’s predictions were the primary contributor as compared to the binary scoring, independently explaining 18.0% of the variance (Supplementary Table 1). Next, we investigated whether PentaMind could differentiate cognitive status with the images scored as 1 (pass/normal) by the conventional clinical evaluation. Intriguingly, the PentaMind exhibited a Spearman correlation of 0.32, accounting for 10.0% of the variance in the global cognition score (Fig. 2b and Supplementary Table 2). The PentaMind accounted for more variance (24%) in a group of failed pentagons (score = 0 from binary scoring). However, according to a linear regression analysis with an interaction term for the two groups, there was no significant difference in the variance explained between the groups (*p*-value = 0.41).These findings demonstrate that our PentaMind can capture nuanced characteristics of human drawings that are undetectable, unquantifiable, or omitted by the conventional clinical assessment.

### Relationship of PentaMind’s prediction with age and education

Age and education level are well-known factors that influence cognitive function. Therefore, it is important to investigate the extent to which these elements affect the predictive accuracy of PentaMind. By examining the interplay between age, education, and cognitive scores predicted by PentaMind, we aim to elucidate the potential effects of these demographic variables on the performance of our model.

Our investigations revealed a statistically significant negative Spearman’s correlation of −0.28 between age and the cognitive scores predicted by PentaMind (*p*-value = 8.2 × 10^−182^), accounting for approximately 5.8% of the variance. This suggests an inverse relationship, where PentaMind’s predicted cognitive scores decrease as an individual’s age increases. Conversely, a positive correlation of 0.20 was found between education level and predicted cognitive scores, accounting for approximately 3.0% of the variance. This correlation, which is also statistically significant (*p*-value = 7.3 × 10^−94^), indicates that PentaMind tends to predict higher cognitive scores for individuals with more advanced education.

To adjust for the potential confounding effects of age and education, we included these variables as covariates in a linear model. In this adjusted model, the cognitive scores inferred from the pentagon drawings served as predictors, while the actual cognitive scores were treated as the outcome. The results of this adjustment yielded two significant findings. First, even after controlling for age and education, the cognitive scores derived from the pentagon drawings continued to provide crucial cognitive information, explaining 18.4% of the variance in cognition. This underscores the pivotal role of pentagon drawings in cognitive assessment. Second, the inclusion of age and education in the model accounted for an additional 9.2% of the variance in cognition, for a total of 32.7% of the variance explained. Sex did not improve cognitive variance explained, contributing only 0.17% to the total variance.

### Relationship of PentaMind’s prediction with ethnicity

By leveraging the ethnic diversity of our observational community-based cohorts, we examined the generalizability of our model across ethnicity. To do this, we calculated prediction performance separately for white participants (77.6% of participants) and those identifying as African American (21.6% of participants). The predictive performance based on Spearman’s correlation for the two ethnic groups were 0.42 and 0.43, respectively (Fig. 2c), while the percentage of variance in cognitive score explained by PentaMind for white and African American participants were 23.2% and 24.1%, respectively (Supplementary Table 3). This comparable performance demonstrates that our model may successfully generalize across both white and African American participants.

In our ongoing examination of the relationships between ethnicity, education, and the predictive efficacy of PentaMind, we conducted a further analysis that included these factors.

We used a multivariate regression model that included age as a covariate, PentaMind-derived cognitive scores as predictors, and introduced a three-way interaction term. This term linked predicted cognition, years of education, and ethnicity, with actual cognitive scores as the outcome. This analysis focused specifically on white and African American individuals. Consistent with the results in Fig. 2c, our model did not identify a significant interaction between ethnicity and predicted cognition (estimate = 0.262, *p*-value = 0.104). However, we found a moderately significant negative three-way interaction term (−0.0318) between ethnicity, education, and predicted cognition (*p*-value = 3.22 × 10^−3^). Specifically, the effect of predicted cognition on actual cognition appears to be higher for African Americans with higher levels of education compared to their white counterparts, underscoring the importance of considering these complex relationships when interpreting drawing test results.

### Assessing PentaMind’s ability to distinguish between dementia states

Next, we assessed the discriminatory capacity of PentaMind between non-cognitive impairment (NCI), mild cognitive impairment (MCI), and dementia (DM) and contrasted this against a conventional rating approach. Receiver Operating Characteristic (ROC) curves were employed, with the area under the curve (AUC) serving as a proxy for these tests’ accuracy in differentiating these cognitive states.

**Fig. 3:**
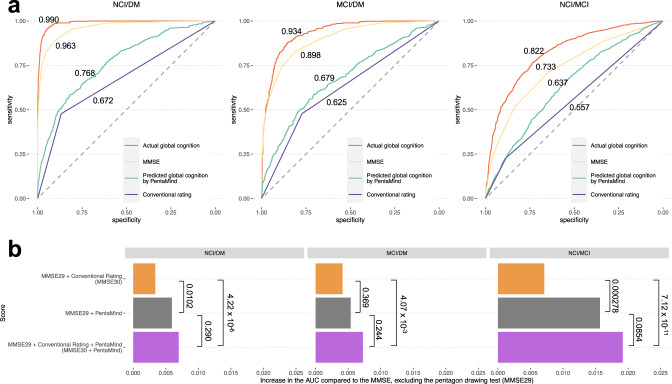
Evaluation and enhancement of dementia assessment with PentaMind. **a** PentaMind’s distinguishing capacity between dementia states. Receiver operating characteristic (ROC) curves show differentiation of non-cognitive impairment (NCI) vs. dementia (DM), mild cognitive impairment (MCI) vs. DM, and NCI vs. MCI. Predictors used were: actual global cognition, MMSE, global cognition predicted by PentaMind, and conventional pentagon drawing rating. Corresponding area under the curve (AUC) values overlay each curve. **b** Improving MMSE performance with PentaMind. Four scores were constructed: the standard MMSE30, MMSE29 (MMSE excluding pentagon drawing test), MMSE29+PentaMind (substituting conventional pentagon drawing rating in MMSE30 with PentaMind), and MMSE30+PentaMind. Incremental AUC improvements over MMSE29 are shown in a bar plot for each contrast: NCI, MCI, and DM. *P*-values displayed were derived using DeLong’s test.

Figure 3a delineates the comparative efficacy of these models. For the NCI versus DM differentiation, PentaMind yielded an AUC of 0.768, exceeding the conventional assessment’s AUC of 0.672 (DeLong’s test, *p*-value < 2.2 × 10^−16^). In distinguishing between MCI and DM, PentaMind displayed an AUC of 0.679, surpassing the conventional scoring with an AUC of 0.625 (*p*-value = 1.6 × 10^−5^). In the distinction between NCI and MCI, PentaMind once more excelled over the conventional rating (*p*-value = 5.2 × 10^−16^), with AUCs of 0.635 and 0.557, respectively.

For comparative analysis, we also examined the actual global cognition and MMSE scores, which constitute composite measures that incorporate multiple tests. As anticipated, global cognition and MMSE outperformed the AUC values across all cognitive contrasts compared to PentaMind and the conventional assessment (Fig. 3a). It’s important to note that dementia diagnosis definition relied on five domain scores within the global cognition score. This likely explains its extremely high accuracy in distinguishing cognitive status.

Collectively, these results highlight two critical findings. Firstly, the PentaMind consistently surpasses the conventional PDT in differentiating between different cognitive states, indicating that PentaMind’s integration may enhance the precision in discerning these states over the traditional rating method. Secondly, despite PentaMind’s promise, current composite measures, such as global cognition and MMSE scores, remain the most precise instruments for distinguishing between NCI, MCI, and DM.

### Enhancing MMSE performance with PentaMind

Given the promising discriminative capacity of PentaMind over the conventional rating approach in differentiating between NCI, MCI, and DM, we sought to determine whether incorporating PentaMind into the MMSE could further improve this accuracy. To this end, we conducted ROC analysis with four scores: the MMSE30 (the standard 30-item MMSE score), the MMSE29 (the MMSE score without the PDT), the MMSE29+PentaMind (replacing the conventional rating of the pentagon drawing in the MMSE30 with PentaMind), and the MMSE30+PentaMind.

Our analysis first examined the differential contributions between conventional rating and PentaMind (Fig. 3b). Interestingly, the AUC of the MMSE29 + PentaMind consistently exceeded that of the MMSE30 across all cognitive state contrasts between NCI, MCI, and DM. Of particular note were the discriminations between NCI and DM and NCI and MCI, where the MMSE29+PentaMind significantly outperformed the MMSE30 with statistically significant AUC differences of 0.003 (DeLong’s test, *p*-value = 0.010) and 0.009 (*p*-value < 0.001), respectively.

Next, we evaluated the influence of PentaMind on the MMSE30 score. The MMSE30+PentaMind consistently produced higher AUCs than the MMSE30 across all contrasts. Specifically, the AUC differences were statistically significant: 0.003 for MCI/DM (*p*-value = 0.004), 0.004 for NCI/DM (*p*-value < 0.001), and 0.012 for NCI/MCI (*p*-value < 0.001). These figures signify a significant enhancement in discriminatory capacity by integrating PentaMind into the MMSE30. Although these increments may seem small at first glance, the practical implications of this improvement are profound, as the inclusion of PentaMind transformed the PDT into the most impactful component for differentiating NCI and MCI among the 30 MMSE items (Supplementary Fig. 2).

Lastly, we evaluated the contribution of conventional scoring to the MMSE29+PentaMind score. Although we observed slight performance improvements, these were not large enough to reach statistical significance.

In summary, our results corroborate the superior performance of PentaMind over the conventional rating in differentiating between cognitive states again. More importantly, we show that incorporating PentaMind into the MMSE30 significantly enhances its discriminatory capacity, particularly in distinguishing between NCI and MCI. Consequently, including PentaMind in the MMSE30 could improve its accuracy in differentiating cognitive states.

### Relationship of PentaMind’s prediction with clinical phenotypes

Global cognition is a summary representation of various aspects of cognitive functions. To understand whether the improvement of PentaMind over the conventional clinical assessment is attributed to the model’s ability to capture specific cognitive components, we analyzed various phenotypic measurements acquired simultaneously from the same individual. Specifically, we explored the link of the predicted global cognition with five domains of global cognition and ten motor functions that are known to be associated with cognitive impairment (Supplementary Table 4). Motor function is a complex phenotype that may necessitate the use of many clinical instruments to capture the various deficiencies that appear in older adults. Therefore, we investigated two interrelated motor phenotypes, a global parkinsonism score and a global motor score, and their domains.

**Fig. 4:**
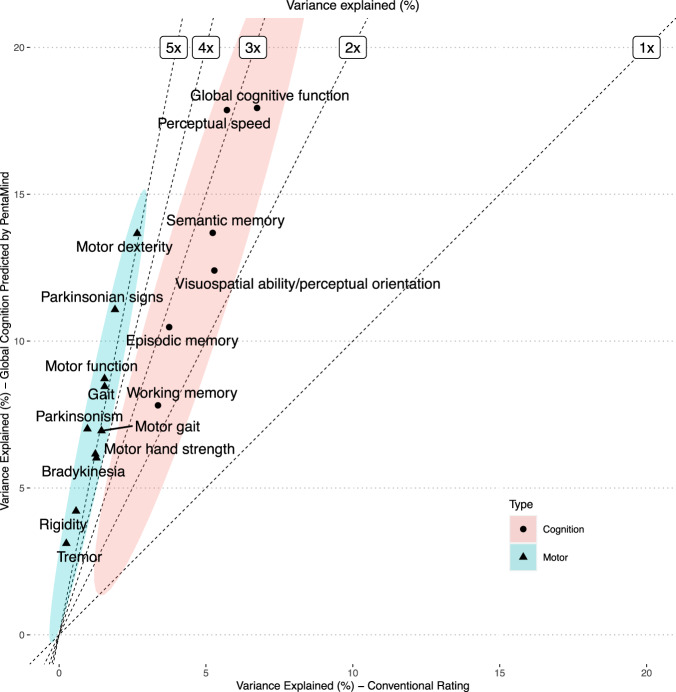
Relationship of Pentamind’s prediction with clinical phenotypes. The percent of the variance in each clinical phenotype explained by the predicted cognition (y-axis), and the conventional clinical rating of PDT (x-axis) is compared. Dashed lines indicate the fold improvements in percent of variance explained by the PentaMind over the conventional rating.

All 15 phenotypes were significantly associated with global cognition score predicted by PentaMind as determined by linear regression analysis (Bonferroni-corrected *p*-value < 0.05). However, only eight were associated with the conventional binary scoring (Fig. 4 and Supplementary Table 5). Overall, PentaMind accounted for about five times more variance than the manual binary scoring alone. Notably, this tendency was more pronounced for motor-related phenotypes than for cognition domains, where the predicted score of PentMind was significantly more strongly associated with motor phenotypes than the manual binary scores, with a magnitude of 6.4 times. While for cognitive scores, the predicted score was also more strongly associated than the manual binary scores, the effect size was smaller, with a magnitude of 2.6 times. The result implies that PentaMind’s improved performance is partly due to its capability to extract signals pertaining to motor impairment from a handwriting image that may be associated with global cognition. The result also highlights the limitation of the conventional scoring, which is dependent on basic indicators such as the number of vertices and the presence of the intersection of two pentagons.

### Relationship of PentaMind’s prediction with brain pathologies

The existence of brain pathologies is the leading cause of cognitive impairment. Consequently, it is important to determine if the global cognition score predicted by the pentagon represents cognitive impairment tied to specific brain pathologies. Therefore, we associated a broad spectrum of brain pathologies, including classical AD pathologies, Lewy bodies, TDP-43, and cerebrovascular pathologies, with the global cognition score predicted by PentaMind based on the PDT closest to death (Supplementary Table 6).

**Fig. 5:**
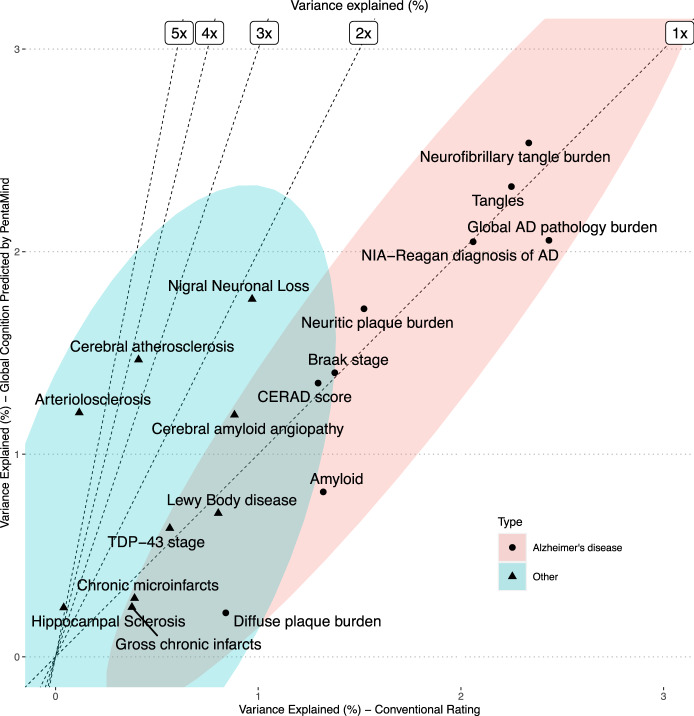
Relationship of Pentamind’s prediction with brain pathologies. The percent of the variance in each brain pathology explained by the predicted cognition and the conventional clinical rating of PDT is compared. Dashed lines indicate the fold improvements in percent of variance explained by the PentaMind over the conventional rating.

Out of 20 pathologic indices, nine were associated with the predicted global cognition as determined by linear regression analysis (Bonferroni-corrected *p*-value < 0.05). Six were associated with the conventional binary score (Fig. 5 and Supplementary Table 7). Comparable effect sizes for classical AD pathologies, including NIA-Reagan and global AD pathology, were observed between the predicted global cognition by PentaMind and the conventional score. PentaMind accounted for 2.05% and 2.06% of the variance in these pathologies, respectively, while the conventional rating explained 2.06% and 2.43%. The Spearman’s correlations for PentaMind were 0.060 and −0.056, compared to 0.075 and −0.086 for the conventional rating.

By contrast, only PentaMind’s prediction showed statistically significant associations with cerebrovascular disease, specifically vessels disease of atherosclerosis and arteriolosclerosis, as well as loss of pigmented neurons in the substantia nigra. Specifically, it accounted for 1.47% of the atherosclerosis variance, 1.21% of arteriolosclerosis, and 1.77% of the loss of pigmented neurons in the substantia nigra, all with respective negative Spearman’s correlations of −0.095, −0.077, and −0.058. In comparison, these pathologies contributed significantly less to the variance in the conventional rating, with values of 0.41%, 0.12%, and 0.97%, respectively.

Since PentaMind appeared to have an advantage in detecting motor-related cognitive impairment, as outlined above, these pathologies might be tied to their known effects on motor function^15^. To examine this hypothesis, we contrasted motor dexterity with the 20 brain pathologies. We found that the cerebrovascular pathologies of atherosclerosis and arteriolosclerosis and nigral neuronal loss were more robustly associated with motor dexterity than the other classical AD pathologies (Supplementary Table 8). These congruent clinical and pathological associations indicate that the deep learning approach can detect the signs of motor dysfunction from pentagon drawing.

### Identification of crucial drawing features

Although PentaMind improved performance in predicting global cognition from the pentagon drawing, elucidating the drawing features contributing to this accuracy is crucial for gaining novel clinical insights. To pinpoint vital elements within the image, we utilized DeepSHAP^16^, a technique for comprehending how a model generates its predictions (Supplementary Fig. 3). DeepSHAP indicated that the model penalizes the poor shape of the pentagon and the lack of well-formed interlocking pentagons. However, it was challenging to specify key drawing features from the result of SHAP. Therefore, we developed a simulator to generate intersecting pentagons with specified parameters. By providing the simulated images to PentaMind and monitoring the predicted cognition values, we were able to identify influential drawing features linked to cognition. To generate a synthetic pentagon drawing, the simulator takes eight parameters, including (1) the number of vertices, (2) the distance between pentagons, (3) alignment of two pentagons, (4) angle distortion, (5) size equality, (6) pentagon size, (7) line width, and (8) line waviness.

**Fig. 6:**
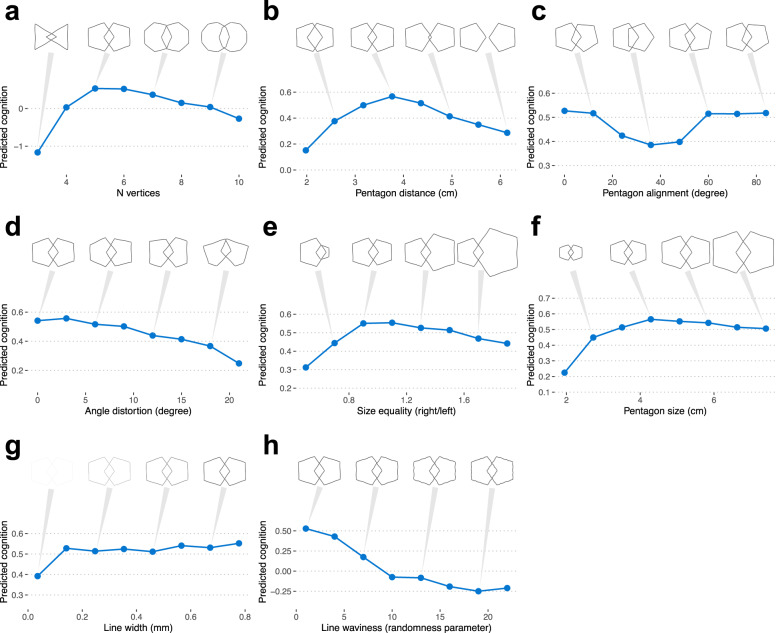
Identification of crucial drawing features. The effect of eight drawing attributes on predicted cognition sore is examined using synthetic images of PDT. The eight drawing attributes include (**a**) the number of vertices, (**b**) the distance between pentagons, (**c**) alignment of two pentagons, (**d**) angle distortion, (**e**) size equality, (**f**) pentagon size, (**g**) line width, and (**h**) line waviness. A point represents the median value and the absolute median deviation of 20 images, respectively.

First, we examined the number of vertices and the presence of intersections—two qualities that are evaluated in the conventional clinical assessment (Fig. 6). As the number of vertices in a drawing increases or decreases, PentaMind’s prediction of global cognition score drops, demonstrating that the model accurately recognizes the geometry of the pentagon (Fig. 6a). Additionally, the distance between two pentagons affects the predicted cognition. Consistent with the conventional evaluation, we observed a lower cognition score when no intersection was present (Fig. 6b). However, PentaMind indicated that excessive overlap is also indicative of diminished cognitive ability (Fig. 6b). Furthermore, the parts of pentagons that intersected influenced the prediction. Specifically, the prediction for global cognition score was highest when vertices of the two pentagons overlapped. However, the predicted cognition score was reduced when one pentagon’s vertex intersected the other’s side (Fig. 6c). This is noteworthy given that in the conventional assessment, any sort of overlap is a prerequisite for pass of the PDT. Nonetheless, PentaMind model dissects the overlaps with respect to cognitive performance in greater detail. This demonstrates how PentaMind can quantify nuanced drawing features.

With the aid of PentaMind, we further explored key drawing features associated with cognition, including the shape and size of the pentagons. As anticipated, PentaMind assigned a higher cognition score to regular pentagons—a five-sided polygon in which all sides and angles are equal (Fig. 6d). The proportionality of the size of the two pentagons was also correlated with a higher predicted cognitive score (Fig. 6e). We observed a sharp decline in the prediction cognition when the size of the drawing was reduced (Fig. 6f), which is in line with the fact that micrographia often seen in Parkinson’s disease^17^. Regarding line width, the cognition score decreased when the line was too thin but had a moderate effect otherwise (Fig. 6g). Notably, the increase in line waviness had a strong influence on the prediction, even if the shape still appeared to be regular pentagons (Fig. 6h). The line waviness likely reflects motor impairment, as drawing a straight line requires proper motor executions. Therefore, the model’s ability to quantify the line waviness may contribute to the enhanced accuracy of detecting motor-related cognitive impairment shown in Fig. 4. This finding suggests line quality as a significant parameter for the cognitive assessment of hand drawing, which the conventional visual inspection mostly ignores.

## Discussion

This work revisited data from a traditional paper-and-pencil drawing test collected for decades with deep learning technology, bringing innovation to healthcare and life science. Results obtained with cutting-edge data analysis outperformed the standard human-based rating by about two-fold for predictions of cognitive function, revealed the novel relationships of hand-drawn pentagons with motor and related brain pathologies, and nominated specific drawing attributes affected by cognitive function. Our approach quantifies the contributions of diverse cognitive and motor neural systems underlying a commonly employed drawing task that can advance our understanding of the well-known association of cognitive and motor decline in older adults.

Our results suggest that quantifying facets of motor function underlying the drawn image was the primary source of the improved model performance compared to the conventional scoring. The subsequent analysis of brain pathologies, including cerebral atherosclerosis, arteriolosclerosis, and nigral neuronal loss, also supported the involvement of motor function. For instance, cerebral atherosclerosis and arteriolosclerosis are associated with dementia and Parkinsonism^18^ by triggering neurovascular dysfunction such as decreased blood flow in the brain and impairment of blood-brain barrier integrity^19,20^. Also, the connection between the loss of nigral dopaminergic neurons and Parkinsonism is well-established^21^. Furthermore, the line waviness greatly impacted the predicted cognition, which also supports the connection between hand-motor execution and cognitive impairment. Recently, digital pen technology has enabled the measurement of intricate graphomotor data and shown its utility for cognitive evaluation^22,23^. Thus, incorporating time-series hand-drawing movement into the deep learning model may further improve the predictive accuracy for cognitive status. The success of this approach in highlighting the importance of quantifying sequential drawing for improved performance suggests that this approach may be useful for the assessment of other conventional motor skills, which currently assess only limited facets of the actual movement and do not even capture the duration of cognitive planning prior to the initial movement. Therefore, the significance of deep learning techniques will become even more crucial in analyzing other more complex behaviors, such as gait, whose 3D features are difficult to quantify during the routine clinical assessment of walking. These efforts may lead to a new lexicon of movement features derived from deep learning analysis that can be further examined using simulation, as was done in the current study.

The emergence of digital tools underscores the need to understand their influence on cognitive assessment, especially when comparing traditional pencil-and-paper methods with digital media. We propose to fine-tune an existing model, originally trained on pencil-and-paper drawings, with images produced by digital devices. This calibration is intended to better understand and compensate for the differences between these two media. Digital tools offer the added benefit of capturing nuanced details such as drawing speed, the presence of tremor, and pen pressure. Such dynamic data contribute to a more robust and comprehensive assessment of cognitive function. However, we also intend to leverage the vast amount of historical data collected from pencil and paper drawings. To optimize the use of this static information, we propose a model capable of inferring subtle motion characteristics from the final image. Consequently, this model can be applied to traditional drawings to extract additional information, thus enhancing the significance of these historical data sets.

We leveraged our cohorts’ ethnic diversity to examine the model’s applicability to white and African American populations. The model performed similarly for both ethnicities, indicating that the model is well-generalized across two ethnicities. This comparable performance may suggest that the number of training samples was adequate to detect signals specific to both white and African American individuals. Alternatively, it could indicate that cognition-related drawing characteristics are not influenced by ethnicity. Clarifying these possibilities will guide the development of a generalized biomarker model based on hand drawings, which warrants further investigation. However, due to the small number of deceased people from the African American population (*n* = 225), the findings on brain pathologies mostly reflect the data from the white population. Therefore, follow-up research is required to confirm the relationships between pentagon drawings and vascular pathologies in the African American population.

One of the challenges of using deep learning models is the need for more interpretability, or the ability to understand which image features are being used by the model to make predictions^24^. This is because deep learning models are often complex and have many layers, making it difficult to understand how the model reaches its conclusions. Various methods have been proposed to analyze an image’s most important parts for making predictions. These methods highlight the areas of an image that contain the most predictive information. Still, they may not explain which specific drawing characteristics are associated with cognitive decline. To address this challenge, we developed a fully parametrized simulator to generate a range of synthetic pentagon images. Then we analyzed the model’s predictions on these images to determine which features are most important for making a prediction. Having successfully identified a set of drawing attributes associated with predicted cognition, our approach could provide a novel way to explore handwritten biomarkers. Our result will serve as a resource to develop complementary computational approaches that automate the extraction of drawing features^25^. By quantifying each drawing attribute, we can examine the correlations between each attribute and the various phenotypes in greater depth.

Rapid and accurate assessment of complex behaviors resulting from diverse neural systems is critical to identifying the underlying neural mechanisms that can be targeted for treatment. By using deep learning technology to analyze handwriting images, it may be possible to design a faster and more accurate method for evaluating cognitive status. This could help healthcare providers more quickly identify individuals at risk for developing dementia and other cognitive impairments, allowing for early intervention and improved outcomes. Additionally, using handwriting samples as a biomarker for cognitive performance could provide insight into the molecular status of the brain, potentially advancing the development of precision medicine for dementia and other conditions. Overall, this study highlights the potential of using deep learning technology in healthcare to improve our understanding of cognitive impairments and to develop more effective treatment strategies.

## Method

### Study cohorts

All eligible participants were enrolled in one of three prospective aging studies at the RADC, the Religious Orders Study (ROS)^26^, the Rush Memory and Aging Project (MAP)^26^, and the Minority Aging Research Study (MARS)^27^). These are prospective analytic community-based cohort studies. As community-based cohorts, the studies are far less susceptible to referral bias, which can introduce substantial sociodemographic, clinical, and genetic variations in patient research. These studies collected ethnic data from participants through self-identification. At the time of enrollment, the average age was 77.4, the average length of education was 15.9 years, 72.9% were female, 77.4% were non-Latino white, and 21.9% were African American. All participants consent to undergo yearly comprehensive clinical examinations. Brain donation at the time of death is a condition of ROS and MAP study entry; it is optional for MARS. An Institutional Review Board of Rush University Medical Center approved all studies and participants gave written informed consent in accordance with the Declaration of Helsinki. As applicable, participants also sign an Anatomical Gift Act (AGA) for brain donation at death.

### Pentagon drawing test administration and pipeline processing

PDT was administered yearly to the participants as a part of items of the MMSE^5^. The MMSE is a 30-item screener for gross cognitive impairment and dementia. It evaluates the severity of cognitive impairment across various cognitive domains. In one section, participants are asked to replicate a sample of intersecting pentagons on paper. The pentagons are then rated 0 (fail) or 1 (pass) based on the presence or absence of intersections and the number of vertices.

To prepare the obtained PDT data for training and testing the PentaMind model, we first converted each pair of intersecting pentagons into a digital image. During this digitization process, random noise can sometimes be introduced, which could distort the true representation of the original hand drawings. To address this, we applied a noise removal step using the OpenCV library, specifically utilizing a morphological operation known as opening, which consists of erosion followed by dilation. This technique effectively enhances the image quality by eliminating noise and reducing variations not intrinsic to the original hand drawings.

Following the noise removal, we used an object detection method based on a C-Support Vector Classification algorithm (https://github.com/ttruty/object-detector) to identify and clip the region containing the intersecting pentagon. On the test set, the accuracy of the pentagon detection was 97.7%. We also conducted a manual evaluation to filter out images without pentagon drawings.

Subsequently, to standardize the image size, the images were padded with a white area around the edge of the pentagon. This process ensured the size of each image was standardized to 500 pixels by 500 pixels, while maintaining the original size of the pentagon. Through these steps, we prepared our data for further processing and analysis.

### Cognitive assessments

Each participant underwent comprehensive clinical evaluations at baseline and at each annual follow-up^28^. In summary, the cognitive battery includes 21 cognitive performance tests, 19 of which are used to develop a global composite measure of cognitive function (global cognition score) and 17 of which assess relatively dissociable cognitive domains, including episodic memory (7 measures), semantic memory (3 measures), working memory (3 measures), perceptual speed (2 measures), and visuospatial ability (2 measures).

A clinical diagnosis of cognitive status involves a three-step process. First, participants take 19 computer-scored cognitive tests that provide severity ratings for five cognitive domains. Next, a neuropsychologist reviews these scores and other clinical data and judges the presence and degree of cognitive impairment. Finally, a clinician evaluates all the data and examines the participant, providing a final diagnostic classification. The diagnosis of dementia and Alzheimer’s disease follows NINCDS/ADRDA guidelines. If a participant is found to have a cognitive impairment but doesn’t meet the criteria for dementia, they are diagnosed with MCI.

### Motor assessments

Each participant was scored with two motor-related assessments: a global parkinsonism score and a global motor score. The global parkinsonism score was calculated by averaging the scores from each of the four parkinsonian domains^26^, which include bradykinesia, tremor, rigidity, and parkinsonian gait. The parkinsonian domains were assessed by qualified nurse clinicians using a modified version of the United Parkinson’s Disease Rating Scale (UPDRS)^29,30^. A higher score suggests more severe parkinsonian impairment of motor function. The global motor score is a summary of ten motor tests from four categories (1) hand strength (two items), (2) motor gait (four items), (3) motor dexterity (two items), and (4) motor balance (two items)^31^. To retain consistency with the global parkinsonism score, the sign of the global motor score and its components has been inverted such that a higher score reflects a more severe motor impairment. These two scores are independently linked with worse health outcomes when evaluated together^32^.

### Neuropathologic evaluations

We generated continuous measures for neuritic plaques, diffuse plaques, and neurofibrillary tangles. The modified NIA-Reagan criteria for diagnosing Alzheimer’s disease comprise the CERAD score for neuritic plaques and the Braak stage for neurofibrillary tangles^33^. Global AD pathology burden is a quantitative summary of AD pathology derived from counts of three AD pathologies: neuritic plaques, diffuse plaques, and neurofibrillary tangles, as determined by microscopic examination of silver-stained slides from five regions. For molecular specificity, we also quantified the load of parenchymal deposition of β-amyloid, and the density of abnormally phosphorylated paired helical filament tau (PHFtau)-positive neurofibrillary tangles, as previously described^34^. Additionally, we evaluated the extent of nigral neuronal loss, the presence of Lewy bodies^35^, the TDP-43 staging^36^, hippocampal sclerosis^37^, chronic macroscopic and microinfarcts^38^, cerebral amyloid angiopathy (CAA)^39^, the severity of atherosclerosis^40^, and arteriolosclerosis^40^.

### Model training

The convolutional neural network, a type of deep learning model, excels in image recognition tasks. Its architecture incorporates a convolutional layer that extracts features from the input image using learned filters, which scan the image for meaningful patterns and features. The output of the convolutional layer then goes through a series of fully connected layers for the final classification or regression task.

For our specific application in predicting global cognition from pentagon drawings, we used 47 vision recognition models provided by Torchvision (v0.11.1), a Pytorch-based library offering various model architectures and pre-trained weights for computer vision. We modified the final layer of these model architectures to output a single numeric value representing the global cognition score.

To initialize the model parameters, we used pre-trained weights from ImageNet^14^. Further fine-tuning was performed with a stochastic gradient descent (SGD) optimizer, configured with specific learning rates, momentum, weight decay, and batch size parameters. For AlexNet and SqueezeNet, we used a different learning rate. Our images were resized to 224 pixels by 224 pixels, and we set the maximum number of training epochs at 90, with an early termination threshold based on validation loss.

To enhance the robustness of our model and to reduce the chances of overfitting, we employed a comprehensive data augmentation strategy with imgaug (version 0.4.0)^41^. This involved applying a range of transformations to the input images. These transformations included flipping, rotation, affine transformations (which involve translating and rotating the images), as well as adjustments in contrast, brightness, and sharpness. Additional transformations, such as adding Gaussian noise or salt-and-pepper noise, were also selectively applied. These transformations did not introduce random noise but rather controlled, systematic changes that help the model generalize better across various scenarios and conditions. The order of these transformations was randomized, and the operations were performed in a sequential manner. This strategy ensured a diverse set of augmented images for training, enabling our model to handle a broad range of data variations effectively.

A similar data augmentation approach is applied during the prediction phase. Each image is subjected to systematic alterations, generating 30 variations per image. Subsequently, the model generates predictions for each variation and averages the results. This process effectively mitigates any potential bias from minor image quality differences that might persist after noise removal.

All these processes were managed with the workflow system Snakemake^42^ and executed on the Google Cloud Platform using an NVIDIA Tesla T4 GPU.

### ROC analysis

We used the pROC library^43^ to perform ROC analysis to evaluate the discriminative ability of PentaMind across different cognitive statuses, namely NCI, MCI, and DM. To categorize the participants into their respective cognitive groups, we used the clinical diagnosis of cognitive status. Specifically, participants were grouped into NCI, MCI, and DM based on this clinical diagnosis. To evaluate PentaMind’s ability to detect early stages of MCI or DM, we used data from the first diagnosed visits for the MCI and DM groups, and baseline data for the NCI group. This approach allowed us to assess PentaMind’s ability to identify individuals with MCI or DM at an early stage. The ROC curve for each metric was then constructed, and the AUCs were compared using DeLong’s test for two correlated ROC curves, which provided associated *p*-values.

### Incorporating PentaMind into the MMSE30

We aimed to incorporate PentaMind, a predictor of continuous global cognition scores, into the MMSE. However, there are inherent differences in scale and distribution between the PentaMind score and the MMSE score. Specifically, the MMSE uses a binary scoring system, assigning either a 0 or a 1 to each item, in contrast to PentaMind’s continuous global cognition estimates. Consequently, simply adding the PentaMind score to the MMSE score would not be appropriate because of these differences. To address this issue, we used logistic regression to model the cognitive diagnosis using PentaMind’s output. This allowed us to transform the PentaMind output into a probability ranging from 0 to 1 that could be effectively integrated with the MMSE score. To ensure the accuracy and integrity of the probability estimates, we used an out-of-fold prediction strategy. This strategy involved strictly separating the data used to optimize the logistic regression model from the data to which the logistic regression model was applied. After converting PentaMind’s output into probability estimates, we added these values to the corresponding MMSE scores. We then examined the discriminative power of the combined PentaMind-MMSE score using ROC analysis. This analytical method allowed us to assess the ability of our integrated measure to discriminate between different cognitive states.

### Pentagon simulation

We developed a simulator for drawing intersecting pentagons using the Matplotlib visualization library. In this simulation study, we examined eight drawing attributes, including angle distortion, line waviness, line width, the number of vertexes, alignment of two pentagons, the distance between pentagons, pentagon size, and size quality. For each parameter, we tested eight values that deviated from the standard pentagons. For each value of the main parameter tested, we generated 20 images with slight variations by randomly varying the rest of the parameters in a small range around the regular pentagons. A reproducible code and generated images are available at https://github.com/stasaki/PentaMind/. Generated images were supplied to the ten deep learning models, each of which had been trained with a distinct subset of data, and the predicted global cognition scores from the ten models were then averaged.

### Statistical analyses

To examine associations of the pentagon scores with clinical parameters and pathologies, we used Spearman’s correlation and linear regression as appropriate. For a multivariable linear regression model, the proportion of the variance explained by each variable was computed using the variance decomposition approach developed by Chevan and Sutherland^44^, implemented in the relaimpo R package^45^.

### Reporting summary

Further information on research design is available in the Nature Research Reporting Summary linked to this article.

### Supplementary information

Supplemental Material

Reporting Summary

## Data Availability

Images and their associated labels can be requested from the RADC Resource Sharing Hub at www.radc.rush.edu, and under the terms of the data usage agreement, they can be shared.

## Code availability

The trained model and the PDT simulator are available at https://github.com/stasaki/PentaMind/.
