# Supplementary material for: Explainable deep learning approach for extracting cognitive features from hand-drawn images of intersecting pentagons
Source: NPJ Digit Med. 2023 Aug 23;6:157. doi: 10.1038/s41746-023-00904-w (PMC10447434; doi:10.1038/s41746-023-00904-w)
Supplement: Supplementary file 1 — Supplemental Material [file 41746_2023_904_MOESM1_ESM.pdf]

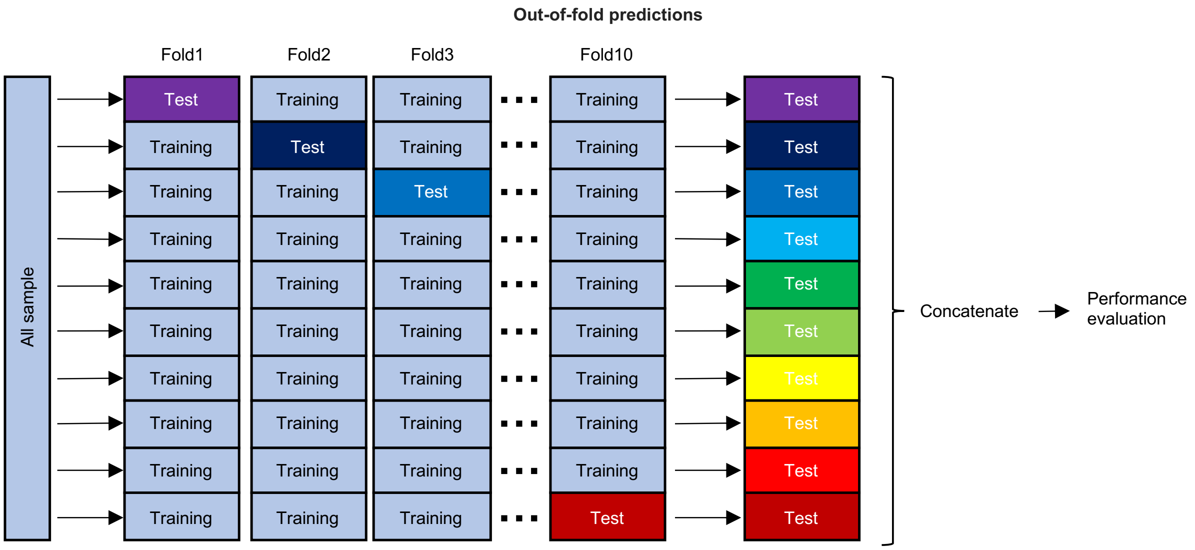

Supplementary Figure 1. The procedure of out-of-fold predictions. We split 3,111 participants into 10 groups, selecting one group as the holdout test set. We then used data from the remaining groups to train PentaMind and generate a prediction for the test set. We repeated this process for all 10 folds, resulting in out-of-fold predictions for all 13,777 images from the 3,111 participants. Finally, we evaluated the performance of the model by comparing predicted cognition scores with actual values.

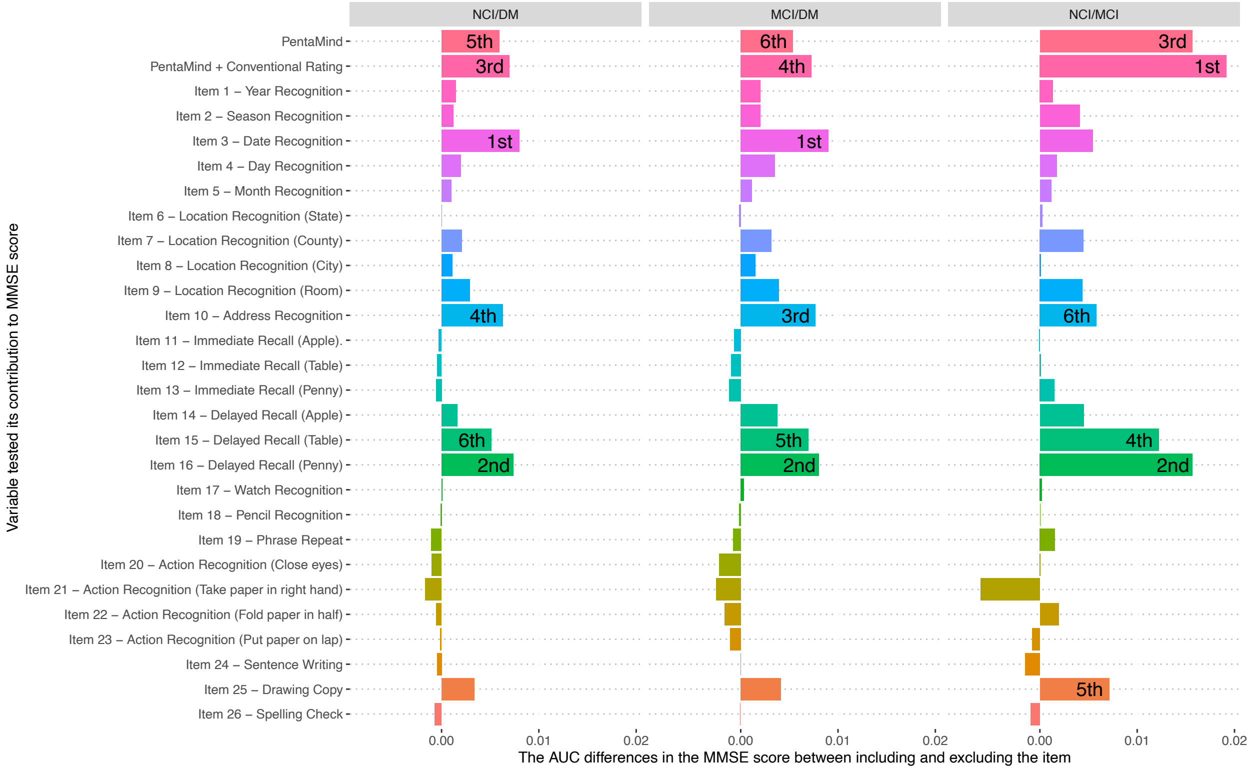

Supplementary Figure 2. Item-level contributions to the MMSE's ability to distinguish between NCI, MCI, and DM. This figure illustrates the contribution of each item on the MMSE to its ability to differentiate between non-cognitive impairment (NCI), mild cognitive impairment (MCI), and dementia

(DM). Individual item contributions were calculated by assessing the differences in the area under the curve (AUC) of the ROC curve when each specific item was included versus excluded from the MMSE score.

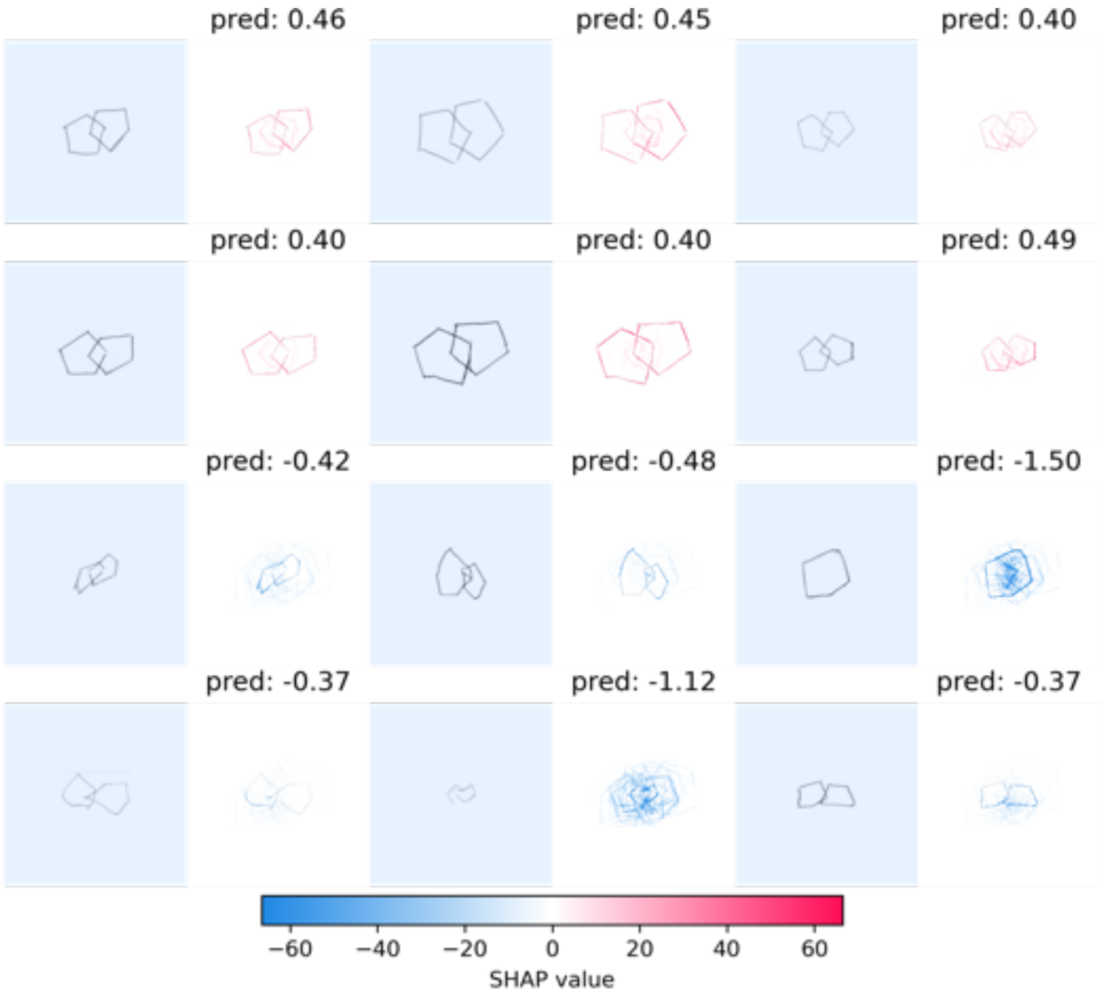

Supplementary Figure 3. DeepSHAP analysis that visualizes the elements influencing prediction. We applied the DeepSHAP method to 6 pentagon drawings from non-cognitively impaired participants and 6 pentagon drawings from cognitively impaired participants. The red color indicates the parts that contribute to higher predicted cognition scores, while the blue color indicates the parts lowering the predicted cognition scores.

Supplementary Table 1. Relationship between predicted cognition scores and actual scores, compared with manual binary ratings.

			linear regression				spearman correlation	
model	**term**	**nobs**	**beta**	**std erro**	**p-value**	**variance explained**	**estimate**	**p-value**
univariate model	predicted global cognition	13747	1.079	0.017	$<2.2\text{x}10^{-16}$	23.3%	0.42	$<2.2\text{x}10^{-16}$
	conventional binary score	13747	0.667	0.015	$<2.2\text{x}10^{-16}$	12.1%	0.30	$<2.2\text{x}10^{-16}$
joint model	predicted global cognition	13747	0.923	0.019	$<2.2\text{x}10^{-16}$	17.9%	-	-
	conventional binary score	13747	0.260	0.017	$<2.2\text{x}10^{-16}$	6.7%	-	-

Supplementary Table 2. PentaMind's performance stratified by the conventional binary rating.

			linear regression				spearman correlation	
**Sample group**	**term**	**nobs**	**beta**	**std error**	**p-value**	**variance explained**	**estimate**	**p-value**
Conventional binary score: 0	predicted global cognition	2483	0.939	0.034	$<2.2\text{x}10^{-16}$	23.6%	0.46	$<2.2\text{x}10^{-16}$
Conventional binary score: 1	predicted global cognition	11264	0.907	0.026	$<2.2\text{x}10^{-16}$	10.0%	0.32	$<2.2\text{x}10^{-16}$

Supplementary Table 3. PentaMind's performance stratified by ethnicity.

			linear regression				spearman correlation	
**Sample group**	**term**	**nobs**	**beta**	**std error**	**p-value**	**variance explained**	**estimate**	**p-value**
white individuals	predicted global cognition	2063	1.110	0.019	$<2.2\text{x}10^{-16}$	23.2%	0.42	$<2.2\text{x}10^{-16}$
African Americans individuals	predicted global cognition	11600	0.883	0.034	$<2.2\text{x}10^{-16}$	24.2%	0.43	$<2.2\text{x}10^{-16}$

Supplementary Table 4. Summary of cognitive and motor phenotypes in the ROS, MAP, and MARS cohorts.

	**ROS**	**MAP**	**MARS**
Number of participants	1093	1508	510
Number of samples	5575	6849	1353
Global cognitive function (mean (SD))	-0.01 (0.73)	-0.11 (0.73)	-0.13 (0.57)
Episodic memory (mean (SD))	0.10 (0.88)	-0.10 (0.88)	-0.03 (0.68)
Visuospatial ability/perceptual orientation (mean (SD))	-0.11 (0.77)	0.10 (0.77)	-0.32 (0.74)
Perceptual speed (mean (SD))	-0.04 (0.93)	-0.20 (0.95)	-0.19 (0.86)
Semantic memory (mean (SD))	-0.14 (0.89)	-0.11 (0.88)	-0.13 (0.78)
Working memory (mean (SD))	0.02 (0.73)	-0.06 (0.73)	-0.21 (0.67)
Parkinsonian signs (mean (SD))	10.46 (7.71)	10.38 (8.24)	5.01 (5.36)
Bradykinesia score (mean (SD))	12.14 (10.30	12.63 (12.26	8.69 (11.64)
Rigidity score (mean (SD))	5.00 (7.93)	3.96 (7.67)	0.53 (2.56)
Gait score (mean (SD))	20.78 (16.54	21.47 (17.45	10.06 (12.45)
Tremor score (mean (SD))	3.10 (4.56)	2.67 (4.89)	0.87 (3.33)
Parkinsonism (mean (SD))	1.03 (0.68)	1.03 (0.74)	0.58 (0.71)
Motor function (mean (SD))	0.98 (0.25)	0.93 (0.23)	1.08 (0.21)
Motor dexterity (mean (SD))	0.97 (0.18)	0.95 (0.18)	1.03 (0.18)
Motor gait (mean (SD))	0.98 (0.26)	0.97 (0.25)	1.02 (0.21)
Motor hand strength (mean (SD))	0.97 (0.29)	0.86 (0.26)	1.10 (0.30)

Supplementary Table 5. Relationships between predicted cognition scores and manual binary ratings with cognitive and motor phenotypes. The associations were determined using a linear regression model.

Type	Clinical phenotype	Term	Estimate	Std.error	Statistic	P-value	Variance explained (%)
Cognition	Global cognitive function	predicted global cognition	0.922855536	0.019286161	47.85066074	0	17.93801344
		conventional clinical score	0.259553803	0.01653785	15.6945314	4.95E-55	6.739572986
	Episodic memory	predicted global cognition	0.901870716	0.026190727	34.43473341	2.91E-249	10.47649366
		conventional clinical score	0.236258914	0.022160722	10.6611559	1.96E-26	3.74740278
	Visuospatial ability/perceptual orientation	predicted global cognition	0.893089417	0.023892232	37.37990723	3.57E-291	12.40429812
		conventional clinical score	0.298936178	0.020034534	14.92104485	5.98E-50	5.285847867
	Perceptual speed	predicted global cognition	1.269972125	0.026308998	48.27139866	0	17.86685591
		conventional clinical score	0.283173286	0.022282794	12.70815897	8.61E-37	5.713254154
	Semantic memory	predicted global cognition	0.979592546	0.024371838	40.19362668	0	13.68589344
		conventional clinical score	0.282090802	0.020898809	13.49793668	2.95E-41	5.229776365
	Working memory	predicted global cognition	0.636388526	0.022321917	28.50958189	9.88E-174	7.8128883
		conventional clinical score	0.21520978	0.019153075	11.2363045	3.63E-29	3.36640125
Motor	Parkinsonian signs	predicted global cognition	-8.72368935	0.256967044	-33.94866987	2.95E-240	11.0739754
		conventional clinical score	-0.252434126	0.218997511	-1.15268034	0.249066839	1.902497848
	Bradykinesia	predicted global cognition	-9.563146904	0.40225126	-23.77406327	7.50E-122	6.021601821
		conventional clinical score	-0.823943935	0.341588912	-2.412092155	0.015877662	1.270364837
	Rigidity	predicted global cognition	-5.545534215	0.271459512	-20.42858685	4.64E-91	4.212688264
		conventional clinical score	0.163933823	0.23149675	0.708147404	0.478868843	0.577448096
	Gait	predicted global cognition	-17.62202226	0.611481051	-28.81859092	5.24E-176	8.450877494
		conventional clinical score	-0.946383154	0.516925969	-1.830790501	0.067159388	1.550305924
	Tremor	predicted global cognition	-2.914146294	0.15716936	-18.54144025	1.37E-75	3.109981386
		conventional clinical score	0.600916379	0.134154787	4.47927645	7.57E-06	0.24604016
	Parkinsonism	predicted global cognition	-0.721103051	0.026894627	-26.81216018	2.00E-153	7.011711404
		conventional clinical score	0.021400885	0.02294997	0.932501652	0.351097937	0.964768375
	Motor function	predicted global cognition	0.261339831	0.008654025	30.19864527	9.45E-193	8.722391136
		conventional clinical score	0.017577844	0.007204669	2.439785179	0.014711226	1.547071493
	Motor dexterity	predicted global cognition	0.230102689	0.005470679	42.0610842	0	13.66489434
		conventional clinical score	0.015834599	0.004651209	3.404404718	0.000665033	2.658677117
	Motor gait	predicted global cognition	0.262097665	0.009636983	27.19706525	3.74E-158	6.946650827
		conventional clinical score	0.027969892	0.008085543	3.459247242	0.000543551	1.443930789
	Motor hand strength	predicted global cognition	0.242414285	0.010760305	22.52857016	1.57E-109	6.154547791
		conventional clinical score	0.016824415	0.009138369	1.841074195	0.065641881	1.234692224

Supplementary Table 6. Summary of brain pathology measurements in the ROS, MAP, and MARS cohorts.

	ROS	MAP	MARS
Number of participants	701	758	28
Age death (mean (SD))	88.72 (6.77)	90.51 (6.08)	84.22 (7.60)
Braak stage (mean (SD))	3.61 (1.24)	3.66 (1.23)	3.43 (1.62)
CERAD score (mean (SD))	2.20 (1.13)	2.20 (1.14)	2.14 (1.24)
NIA-Reagan diagnosis of AD (mean (SD))	2.13 (0.75)	2.17 (0.74)	2.21 (0.96)
Global AD pathology burden (mean (SD))	0.76 (0.62)	0.75 (0.63)	0.69 (0.71)
Neurofibrillary tangle burden (mean (SD))	0.63 (0.73)	0.69 (0.83)	0.76 (1.09)
Diffuse plaque burden (mean (SD))	0.77 (0.79)	0.69 (0.75)	0.42 (0.37)
Neuritic plaque burden (mean (SD))	0.89 (0.86)	0.87 (0.84)	0.88 (0.94)
Amyloid (mean (SD))	3.44 (3.50)	4.59 (4.39)	3.07 (3.38)
Tangles (mean (SD))	7.09 (8.53)	7.58 (8.88)	9.78 (13.94)
Lewy Body disease (mean (SD))	0.59 (1.10)	0.57 (1.10)	0.75 (1.24)
Arteriolosclerosis (mean (SD))	1.06 (0.97)	1.14 (0.92)	0.89 (0.92)
Nigral Neuronal Loss (mean (SD))	0.57 (0.80)	0.55 (0.75)	0.46 (0.74)
Cerebral atherosclerosis (mean (SD))	1.26 (0.79)	1.18 (0.83)	0.75 (0.70)
Cerebral amyloid angiopathy (mean (SD))	1.29 (0.96)	1.27 (0.92)	0.79 (0.96)
Gross chronic infarcts (mean (SD))	0.34 (0.47)	0.37 (0.48)	0.29 (0.46)
Chronic microinfarcts (mean (SD))	0.30 (0.46)	0.31 (0.46)	0.21 (0.42)
TDP-43 stage (mean (SD))	1.02 (1.20)	1.15 (1.24)	1.07 (1.30)
Presence of Lewy bodies in 7 regions (mean (SD))	0.26 (0.44)	0.25 (0.43)	0.29 (0.46)
Definite presence of typical hippocampal sclerosis (mean (SD))	0.08 (0.28)	0.10 (0.30)	0.04 (0.21)
Hippocampal Sclerosis (mean (SD))	0.09 (0.29)	0.11 (0.31)	0.04 (0.21)

Supplementary Table 7. Relationships between predicted cognition scores and manual binary ratings with brain pathology measurements. The associations were determined using a linear regression model.

Type	Pathology	Term	Estimate	Std.error	Statistic	P-value	Variance explained (%)
Alzheimer's disease	Braak stage	predicted global cognition	-0.259892117	0.085473992	-3.040598804	0.002402791	1.402190897
		conventional clinical score	-0.242756338	0.081499261	-2.978632371	0.002942801	1.377539492
	CERAD score	predicted global cognition	0.235262045	0.078256209	3.006305163	0.002689241	1.351202565
		conventional clinical score	0.213597473	0.074617122	2.862579898	0.004261595	1.295377754
	NIA-Reagan diagnosis of AD	predicted global cognition	0.186543581	0.050794164	3.672539676	0.000248753	2.048813216
		conventional clinical score	0.179028658	0.048432122	3.696485913	0.00022663	2.060316238
	Amyloid	predicted global cognition	-0.517814357	0.281340765	-1.840523738	0.065900373	0.814128547
		conventional clinical score	-0.87918222	0.268462964	-3.274873398	0.001082622	1.320876402
	Tangles	predicted global cognition	-2.378515778	0.60898352	-3.90571452	9.83E-05	2.321194546
		conventional clinical score	-2.182451903	0.579674947	-3.764958124	0.000173322	2.249192454
	Global AD pathology burden	predicted global cognition	-0.149008875	0.042953522	-3.469072311	0.000537354	2.055889455
		conventional clinical score	-0.173255795	0.041003916	-4.225347503	2.53E-05	2.434229439
	Neurofibrillary tangle burden	predicted global cognition	-0.227198003	0.05392646	-4.213108035	2.67E-05	2.536576154
		conventional clinical score	-0.196925141	0.051478806	-3.825363393	0.000136051	2.334735339
	Diffuse plaque burden	predicted global cognition	-0.01722381	0.053070029	-0.324548716	0.745568854	0.217051774
		conventional clinical score	-0.154931195	0.050661247	-3.058179641	0.002267098	0.83988327
	Neuritic plaque burden	predicted global cognition	-0.202605484	0.058251032	-3.478144121	0.000519636	1.71804851
		conventional clinical score	-0.167910928	0.055607091	-3.0195956	0.002574771	1.521792762
Hippocampal sclerosis	Hippocampal Sclerosis	predicted global cognition	-0.035935955	0.02103766	-1.708172643	0.087820885	0.242905988
		conventional clinical score	0.001583114	0.020054611	0.078940158	0.937091243	0.040297725
Lewy body/PD	Lewy Body disease	predicted global cognition	-0.156204588	0.077431346	-2.017330128	0.043848257	0.708534743
		conventional clinical score	-0.172667786	0.074071694	-2.331090011	0.019887485	0.802832238
	Nigral Neuronal Loss	predicted global cognition	-0.21018765	0.053137875	-3.955514755	8.00E-05	1.765580825
		conventional clinical score	-0.096829432	0.050718117	-1.909168523	0.056435281	0.969883158
TDP-43	TDP-43 stage	predicted global cognition	-0.178076124	0.087569233	-2.033546697	0.042186806	0.634831623
		conventional clinical score	-0.147579723	0.08335197	-1.770560707	0.076852764	0.563772256
Vascular - General measures	Arteriolosclerosis	predicted global cognition	-0.27698215	0.06551893	-4.227513345	2.51E-05	1.205233291
		conventional clinical score	0.082512673	0.062403909	1.322235646	0.186296513	0.116972324
	Cerebral atherosclerosis	predicted global cognition	-0.226024862	0.05610181	-4.028833684	5.89E-05	1.46676538
		conventional clinical score	-0.028928425	0.053367729	-0.542058386	0.58785994	0.409384793
	Cerebral amyloid angiopathy	predicted global cognition	-0.198967923	0.066088522	-3.0106275	0.002652326	1.194027651
		conventional clinical score	-0.132744763	0.06275561	-2.115265283	0.034578209	0.882793969
Vascular - Infarcts	Gross chronic infarcts	predicted global cognition	-0.034804839	0.033306924	-1.044973084	0.296207681	0.244680635
		conventional clinical score	-0.055436692	0.031788759	-1.743908653	0.081384621	0.376818022
	Chronic microinfarcts	predicted global cognition	-0.038765654	0.03202324	-1.210547519	0.226264	0.289090482
		conventional clinical score	-0.052511392	0.030563587	-1.718103059	0.085988974	0.389797268

Supplementary Table 8. Relationships between motor dexterity and brain pathology measurements. The associations were determined using a linear regression model.

Type	Pathology	Estimate	Std.error	Statistic	P-value	Variance explained (%)	N obs.
Vascular - General measures	Arteriolosclerosis	-0.0388702	0.0058968	-6.5917483	6.16E-11	3.043967261	1386
Vascular - Infarcts	Gross chronic infarcts	-0.0743475	0.01172265	-6.34221	3.06E-10	2.816339633	1390
Vascular - General measures	Cerebral atherosclerosis	-0.0412076	0.00693797	-5.9394249	3.60E-09	2.456126083	1403
Lewy body/PD	Nigral Neuronal Loss	-0.0336266	0.00729753	-4.6079505	4.44E-06	1.505652233	1391
Alzheimer's disease	Diffuse plaque burden	-0.0253828	0.00736655	-3.4456777	0.00058659	0.846313048	1393
Vascular - Infarcts	Chronic microinfarcts	-0.0416423	0.01227183	-3.3933237	0.00070995	0.822759908	1390
Alzheimer's disease	Global AD pathology burden	-0.0229891	0.00901898	-2.5489668	0.01091087	0.464919097	1393
Hippocampal sclerosis	Hippocampal Sclerosis	-0.0478108	0.01951732	-2.4496613	0.01442481	0.440590064	1358
Vascular - General measures	Cerebral amyloid angiopathy	-0.0145305	0.00597161	-2.4332669	0.01509056	0.430627565	1371
TDP-43	TDP-43 stage	-0.0109559	0.00473074	-2.3159001	0.02071679	0.405898415	1318
Alzheimer's disease	Amyloid	-0.0033182	0.00144249	-2.3003279	0.02158267	0.393335451	1342
Alzheimer's disease	CERAD score	0.01052324	0.00495099	2.12548094	0.03372205	0.323263836	1395
Alzheimer's disease	Braak stage	-0.0090888	0.00454776	-1.9985255	0.04585378	0.28590701	1395
Lewy body/PD	Lewy Body disease	-0.0104354	0.00522304	-1.9979455	0.04592319	0.295252264	1350
Alzheimer's disease	NIA-Reagan diagnosis of AD	0.01352468	0.00758731	1.78253999	0.07487895	0.227582018	1395
Alzheimer's disease	Neurofibrillary tangle burden	-0.0111099	0.00714567	-1.5547802	0.1202259	0.173482947	1393
Alzheimer's disease	Tangles	-0.0009217	0.00065188	-1.4139379	0.15761002	0.147435213	1356
Alzheimer's disease	Neuritic plaque burden	-0.0074431	0.00675596	-1.1017038	0.27078107	0.087181383	1393
