# Supplementary material for: Explainable deep learning approach for extracting cognitive features from hand-drawn images of intersecting pentagons
Source: NPJ Digit Med. 2023 Aug 23;6:157. doi: 10.1038/s41746-023-00904-w (PMC10447434; doi:10.1038/s41746-023-00904-w)
Supplement: Supplementary file 2 — Reporting Summary [file 41746_2023_904_MOESM2_ESM.pdf]

nature portfolio

Corresponding author(s): Shinya Tasaki

Last updated by author(s): Jun 12, 2023

Reporting Summary

Nature Portfolio wishes to improve the reproducibility of the work that we publish. This form provides structure for consistency and transparency in reporting. For further information on Nature Portfolio policies, see our Editorial Policies and the Editorial Policy Checklist.

Statistics

For all statistical analyses, confirm that the following items are present in the figure legend, table legend, main text, or Methods section.

n/a	Confirmed	
☐	☒	The exact sample size (*n*) for each experimental group/condition, given as a discrete number and unit of measurement
☐	☒	A statement on whether measurements were taken from distinct samples or whether the same sample was measured repeatedly
☐	☒	The statistical test(s) used AND whether they are one- or two-sided<br>*Only common tests should be described solely by name; describe more complex techniques in the Methods section.*
☐	☒	A description of all covariates tested
☐	☒	A description of any assumptions or corrections, such as tests of normality and adjustment for multiple comparisons
☐	☒	A full description of the statistical parameters including central tendency (e.g. means) or other basic estimates (e.g. regression coefficient) AND variation (e.g. standard deviation) or associated estimates of uncertainty (e.g. confidence intervals)
☐	☒	For null hypothesis testing, the test statistic (e.g. *F*, *t*, *r*) with confidence intervals, effect sizes, degrees of freedom and *P* value noted<br>*Give P values as exact values whenever suitable.*
☒	☐	For Bayesian analysis, information on the choice of priors and Markov chain Monte Carlo settings
☒	☐	For hierarchical and complex designs, identification of the appropriate level for tests and full reporting of outcomes
☐	☒	Estimates of effect sizes (e.g. Cohen's *d*, Pearson's *r*), indicating how they were calculated

Our web collection on statistics for biologists contains articles on many of the points above.

Software and code

Policy information about availability of computer code

Data collection	object-detector v1<br>python v3.9.12<br>opencv-python v4.7.0.72<br>scikit-learn v1.2.2
Data analysis	PentaMind v1<br>relaimpo v2.2-6<br>matplotlib v3.5.0<br>pROC v1.18.2<br>snakemake v5.24.1<br>imgaug v0.4.0<br>torchvision v0.11.1<br>pytorch v1.10.0

For manuscripts utilizing custom algorithms or software that are central to the research but not yet described in published literature, software must be made available to editors and reviewers. We strongly encourage code deposition in a community repository (e.g. GitHub). See the Nature Portfolio guidelines for submitting code & software for further information.

Data

Policy information about availability of data

All manuscripts must include a data availability statement. This statement should provide the following information, where applicable:

- Accession codes, unique identifiers, or web links for publicly available datasets
- A description of any restrictions on data availability
- For clinical datasets or third party data, please ensure that the statement adheres to our policy

The data can be requested at the RADC Resource Sharing Hub at www.radc.rush.edu.

Research involving human participants, their data, or biological material

Policy information about studies with human participants or human data. See also policy information about sex, gender (identity/presentation), and sexual orientation and race, ethnicity and racism.

Reporting on sex and gender	Our research uses ROS, MAP, and MARS, which are prospective, community-based cohort studies. Recruitment of participants was not influenced or restricted by any demographic criteria, including sex. Participants self-reported their sex for our study. Of our participants, 72.9% were female. We didn't observe sex differences in this study.
Reporting on race, ethnicity, or other socially relevant groupings	This study collected racial data from participants through self-identification, offering options such as White, Black or African American, American Indian or Alaska Native, Native Hawaiian or Other Pacific Islander, Asian, Multi-Racial, Other, and an option for those preferring not to answer.
Population characteristics	At the time of enrollment, the average age was 77.4, the average length of education was 15.9 years, 72.9% were female, 77.4% were non-Latino white, and 21.9% were African American.
Recruitment	ROS, MAP, and MARS are prospective analytic community-based cohort studies. As community-based cohorts, the studies are far less susceptible to referral bias, which can introduce substantial sociodemographic, clinical, and genetic variations in patient research.
Ethics oversight	An Institutional Review Board of Rush University Medical Center approved all studies

Note that full information on the approval of the study protocol must also be provided in the manuscript.

Field-specific reporting

Please select the one below that is the best fit for your research. If you are not sure, read the appropriate sections before making your selection.

☒ Life sciences ☐ Behavioural & social sciences ☐ Ecological, evolutionary & environmental sciences

For a reference copy of the document with all sections, see nature.com/documents/nr-reporting-summary-flat.pdf

Life sciences study design

All studies must disclose on these points even when the disclosure is negative.

Sample size	We utilized all samples for which there is both a pentagon drawing and a global cognition measurement available.
Data exclusions	We removed data points that did not correctly scan the paper involving drawing intersecting pentagons.
Replication	We randomly divided 3,111 participants into a training set (80%), a validation set (10%), and a replication set (10%). To improve the reliability of performance evaluation, we repeated the training procedure five times using five distinct sets of training and replication samples.
Randomization	There is no randomization. ROS, MAP, and MARS are community studies. We enroll everyone who doesn't have dementia at the time of enrollment and agrees to annual detailed clinical and cognitive evaluation. All participants with an available sample were used in the analyses.
Blinding	Both the image processing of the pentagon drawing and the evaluation of impairment and dementia were blinded to ensure that participant demographics and other phenotypes did not influence the analysis or decision.

Reporting for specific materials, systems and methods

We require information from authors about some types of materials, experimental systems and methods used in many studies. Here, indicate whether each material, system or method listed is relevant to your study. If you are not sure if a list item applies to your research, read the appropriate section before selecting a response.

Materials & experimental systems

n/a	Involved in the study
☒	☐ Antibodies
☒	☐ Eukaryotic cell lines
☒	☐ Palaeontology and archaeology
☒	☐ Animals and other organisms
☒	☐ Clinical data
☒	☐ Dual use research of concern
☒	☐ Plants

Methods

n/a	Involved in the study
☒	☐ ChIP-seq
☒	☐ Flow cytometry
☒	☐ MRI-based neuroimaging
